# Mechanical Properties of Coal Ash Particle-Reinforced Recycled Plastic-Based Composites for Sustainable Railway Sleepers

**DOI:** 10.3390/polym12102287

**Published:** 2020-10-06

**Authors:** Suhawn Ju, Jinyoung Yoon, Deokyong Sung, Sukhoon Pyo

**Affiliations:** 1 Department of Urban and Environmental Engineering, Ulsan National Institute of Science and Technology (UNIST), 50 UNIST-gil, Ulju-gun, Ulsan 44919, Korea; gn00147@unist.ac.kr; 2 Department of Civil and Environmental Engineering, Pennsylvania State University, University Park, PA 16801, USA; jpy5278@psu.edu; 3 Department of Civil & Railroad Engineering, Daewon University College, 316 Daehak-ro, Jecheon, Chung-Buk 27135, Korea

**Keywords:** municipal plastic waste, coal ash, railway sleeper, mechanical properties, morphology

## Abstract

This experimental research investigates the mechanical properties of municipal plastic waste-based particulate composites reinforced with coal ash (CA), the by-product of thermal power plants, for sustainable railway sleepers. Six series of sustainable composites filled with inorganic mineral fillers, including CA, were prepared by a twin-screw extruder and a compression molding machine. The effect of mix design variables—such as filler type, contents and the particle size of the filler—on mechanical properties—including tensile, compression and flexural properties—and morphology were characterized. The scanning electron microscopy (SEM) was employed to examine the morphology of the composites, which revealed the uniform dispersion of fillers in the polymer matrix. The study results conclude that the recycled plastic-based composite with the addition of CA up to 60% is suitable for railway sleeper applications. This experimental study may provide new insight into the railway applications of the developed composites under service loading conditions including traffic loading and earthquake.

## 1. Introduction and Motivation

As industrialization progresses, the number of industrial by-products and household wastes being disposed of continues to increase worldwide, and the development of technology that can effectively upcycle these wastes is required. Various forms of plastics are used in modern society in tremendous quantities, which causes enormous environmental problems because the chemical structures of most plastics cannot be dismantled by microorganisms, and therefore they are not naturally decomposed in a short period of time. Plastic waste that is not collected through recovery systems covers land and sea and causes direct damage to animals. In addition, due to its relatively inexpensive cost, many countries still use coal-fired power to generate electricity, which is responsible for more than 19% of the electricity in the world [1]. However, this power generation method inevitably produces a substantial amount of coal ash (CA) (or coal combustion residues), and the ashes that are not recycled are being put in landfills [2,3]. For example, according to the American Coal Ash Association, only 54% (24 million tons) of CA in America was used beneficially in 2015 [4]. To cope with these environmental problems, there is a demand for efforts to recycle plastic waste and CA in large quantities.

The manufacturing process for particle-reinforced polymer-based composites is well-established and widely applied to various fields. Different types of fillers have been adapted for use in recycled or virgin plastics with various purposes, including the enhancement of dimensional stabilities and mechanical properties [5,6,7,8]. With the additional advantages of using CA, such as its low cost, light weight and abundance, the application of CA as a filler in polymer-based composites for structural applications has been tried by many research groups. For example, Li et al. [9] found that the combination of 50% fly ash to post-consumer Polyethylene terephthalate (PET) plastic led to a 53% increase in compressive strength. Pardo et al. [10] investigated the deformation and fracture behavior of fly ash-reinforced polypropylene (PP) matrix composites and concluded that using the stiffer filler in the polymer matrix resulted in an increased stiffness and decreased tensile strength of the composites. Yao et al. [7] used circulating fluidized bed combustion fly ashes as a filler after they were coated by stearic acid in polymer composites to increase the toughness of the composites. In addition, Khoshnoud and Abu-Zahra [11] experimentally studied the effect of the particle size of fly ash on the mechanical properties of Polyvinyl chloride (PVC) matrix composites. 

It is necessary to develop sustainable, economic and reliable materials for railway sleepers (or ties) to overcome the many limitations of the materials that are currently being used for sleepers, such as the scarcity of timber, the corrosion of steel and the low durability of concrete under chemical environments [12]. Several types of railway sleepers have been developed using different composite materials as alternatives by mimicking the structural responses of wooden railway sleepers [13,14,15,16,17,18]. For example, a variety of composite materials were developed for railway sleepers in the late 1990s and early 2000s, such as glass-fiber reinforced high-density polyethylene (HDPE) matrix composite, polymer-fiber reinforced HDPE matrix composite, and mineral by-product combined with HDPE matrix composite [19]. Manalo and Aravinthan [20] produced glue-laminated composite sandwich beams made up of glass fiber-reinforced polymer matrix composites for railway turnout sleepers. Khalil [21] developed new composite materials for railway sleepers using E-glass fiber, iron slag and plastic wastes consisting of a mixture of polyethylene, polyester and styrene. Based on a series of mechanical tests, it was concluded that the composites met the minimum requirements of the international railway standards. To the best knowledge of the authors, there has been no reported research on the development of sustainable and economic composite materials for railway sleepers by simultaneously using CA and recycled plastics from municipal waste, which further motivated the work discussed in this paper.

The objective of this study is to develop sustainable alternative composites for use in railway sleepers by effectively reusing municipal plastic waste (MPW) and industrial by-products in large quantities. The characteristics of raw materials were evaluated using several techniques, including thermogravimetric analysis (TGA), X-ray fluorescence (XRF), X-ray diffraction (XRD) and gel permeation chromatography (GPC). The mechanical properties of the designed composites were investigated in terms of tensile, compression and flexural responses and compared with the minimum requirements of the international railway standards. The morphology of the fractured surface of the composites was examined using scanning electron microscopy (SEM).

## 2. Materials and Experimental Methodology

### 2.1. Raw Materials

**Figure 1:**
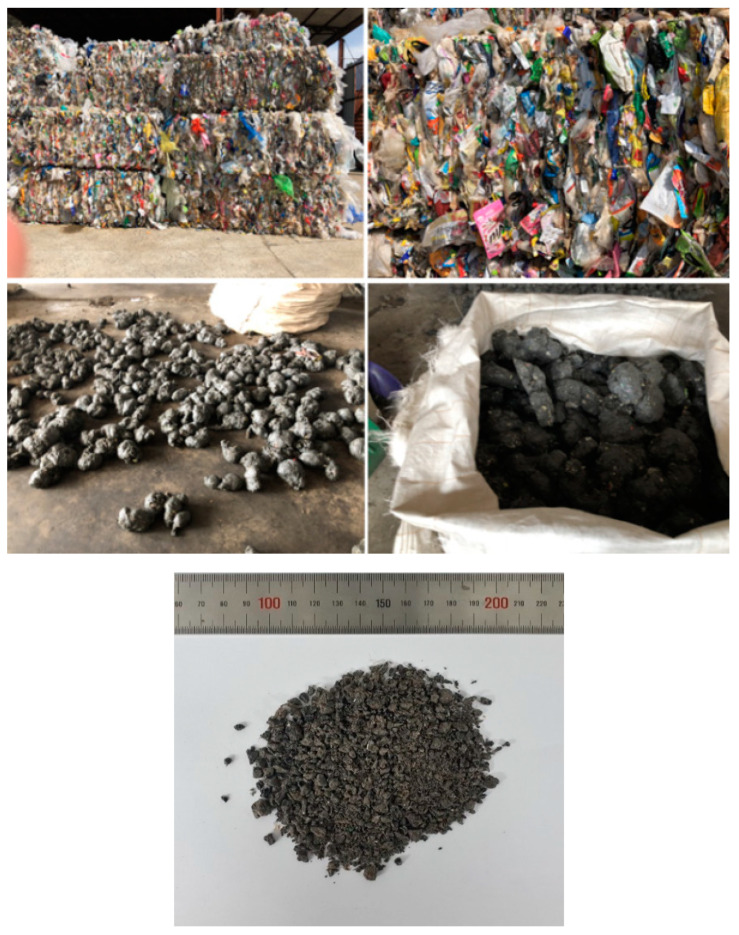
Recycling process of municipal plastic waste (MPW).

**Figure 2:**
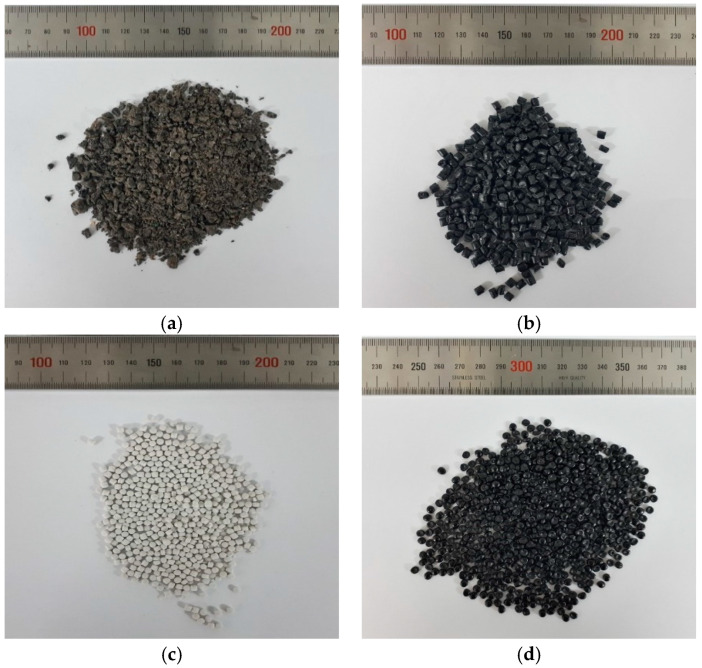
Pelletized raw materials: (**a**) MPW, (**b**) recycled high-density polyethylene (RHDPE), (**c**) calcium carbonate (CaCO_3_) calpet filler (CCF) and (**d**) antistatic agent.

**Figure 3:**
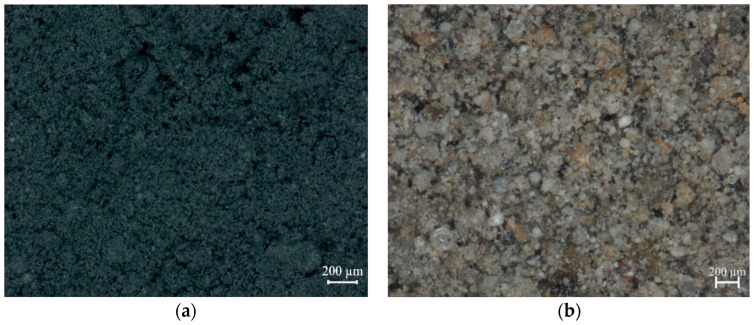

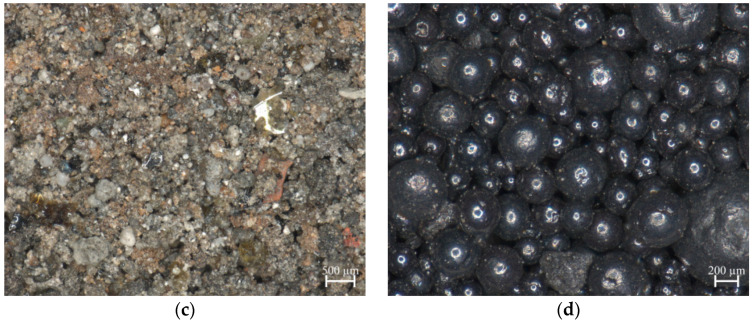
Images of fillers: (**a**) coal ash (CA) I, (**b**) CA II, (**c**) CA III and (**d**) slag aggregates.

The materials utilized as matrix in this study are MPW and recycled HDPE (RHDPE). The MPW consisted of various forms of waste plastics, including PP, low-density polyethylene (LDPE) and HDPE, which were supplied by the Hansung Recycle Co., Sejong, Korea. Figure 1 illustrates the fabrication procedure of MPW pellets from waste plastics. RHDPE is a recycled plastic created using the defective products produced during conventional plastic manufacturing processes and mainly consists of HDPE, which was supplied by Jiju Co., Umseong, Korea. Due to the difference in the source, it is expected that RHDPE has a higher material quality and that it is easier to control the quality of the material compared with MPW. It should be pointed out, however, that MPW and RHDPE were used together as the matrix materials in order to use household wastes and industrial by-products as much as possible while satisfying the requirement for mechanical performances. Figure 2 shows the pelletized raw materials. Furthermore, five different types of fillers were used to evaluate the effectiveness of the fillers, namely, calcium carbonate (CaCO_3_) calpet filler (CCF), three groups of CA with different particle sizes and slag aggregates. Calcium carbonate is the most widely used filler for polymeric composites [22], and the CCF was supplied by Hyo Chang Industry, Seosan, Korea. The CA I was supplied by Korea South-East Power Co., Incheon, Korea. The CA II and III were supplied by Korea East-West Power Co., Yeosu, Korea. In addition, the slag aggregate is a byproduct of steelmaking generated during the refining process and can be classified as basic oxygen furnace (BOF) slag, which was supplied by Yujin Ecociel Co., Dangjin, Korea. Detailed information of the slag aggregate can be found in Koh et al. [23] and Tafesse et al. [24]. Figure 3 shows the fillers used. An antistatic agent was used to lower the resistivity of the composites. Table 1 shows the density of the raw materials used.

### 2.2. Test Methodologies for Raw Materials

The chemical properties of raw plastic materials and fillers were investigated using XRF, XRD, TGA and GPC. The oxide compositions of plastic materials and fillers were characterized using XRF (S8 Tiger, Rigaku, Japan). The crystalline species of CA fillers were investigated by XRD (D/MAX2500 diffractometer, Rigaku, Japan) in a range of 5–60° 2-theta. TGA measures the weight change of a specimen according to the temperature change, which provides qualitative and quantitative evaluation of chemical composites based on a temperature–weight curve. In this study, about 10 mg of raw materials were used for the analysis, and nitrogen (30–800 °C) was heated at a constant rate of 10 °C/min, which was performed on a TGA Q500 system (TA Instruments). 

GPC analysis measures the average molecular weight and weight-average molecular weight of polymers and calculates the polydispersity index (PDI). As plastic is a high-molecular material, GPC analysis is important for the final properties and quality maintenance of polymers. As plastic samples, such as polyethylene or PP, are not soluble at room temperature, high-temperature GPC (HLC-8321 GPC/HT, Tosoh, Japan) was used. The plastic samples were dissolved in the trichlorobenzene solution with 0.04% butylated hydroxytoluene (300 µL). The analysis of molecular weight was conducted at a flow rate of 1 mL/min and with a high temperature of 160 °C, using GPC columns of one PLgel guard (7.5 mm × 50 mm, made by Agilent Technologies) and two PLgel mixed-B columns (7.5 mm × 300 mm, made by Agilent Technologies) with a refractive index detector. The particle size distribution of fillers (i.e., CA and slag aggregates) was determined by a laser diffraction particle size analyzer (LA-950, Horiba, Japan). This equipment provides a wide range measurement for particle sizes (0.01–3000 µm). The particle size obtained from this analysis indicates a volume-equivalent sphere diameter. It should be noted that fillers were oven dried for 24 h before the test to prevent agglomeration of particles. 

### 2.3. Mix Design and Specimen Preparation

The plastic (MPW and RHDPE) pellets and inorganic mineral fillers were blended by dry rotational mixing for 12 h at 110 ± 5 °C prior to compounding. The plastic materials and fillers were melt-blended in a twin-screw extruder (CJWA 75/44, Changzhou JWELL Intelligent Chemical Equipment Co., Liyang, China) of L/D ratio 44:1 and a screw speed of 150 rpm to produce particulate composites. The processing temperature was maintained as 170 °C and 190 °C at the feeder and the header, respectively. Three types of test specimens to meet ISO standards were molded using a compression molding machine (Tae Shin Precision Co., Namyangju, Korea) at a temperature of 160 ± 5 °C and pressure of 50 MPa with a cycle time of 10 min. Subsequently, the molded samples were cooled for 15 min. Although as the content of the fillers increased the viscosity of the mixtures decreased, no major problems were experienced during the extrusion and injection molding process. Table 2 shows the mix proportions of the tested composites. The series #1 to #5 were designed to evaluate the effect of the fillers on the mechanical performances of the recycled plastic-based composites. Meanwhile, series #6 was designed with the aim to be possibly used for railway sleeper applications by adopting the antistatic agent and other additives. It should be noted that the other additives, mainly composed of lubricant and coupling agent, were used in the mixture of series #6 to enhance dispersion and adhesion of fillers because series #6 contains 60% of filler.

### 2.4. Mechanical Tests

A series of mechanical tests were carried out to evaluate the tensile, compressive and flexural performances following ISO standards for plastic materials. The tensile test was conducted following ISO 527-1:2012 [25] to characterize tensile strength, strain at break and modulus. The specimens for tensile tests were prepared according to ISO 527-2:2012 [26] with the gauge length of 75 mm. An axial extensometer was employed to measure elongation of the specimens. The test speed was set to 1 mm/min., according to ISO 527-1:2012 [25]. The compressive test was conducted following ISO 604:2002 [27] to investigate compressive stress at a certain strain value rather than compressive strength because the stress–strain curves achieved in this study did not exhibit a yield point. The test speed was set to 1 mm/min., according to ISO 604:2002 [27]. The three-point flexural test was carried out to evaluate flexural strength of the composites following ISO 178:2010 [28]. The test speed was set to 2 mm/min., according to ISO 178:2010 [28]. The mechanical tests were conducted using a universal testing machine (UTM), and the strength was determined by averaging the tested values of the five replicates. It should be noted that the ISO standard suggested material requirements for railway plastic sleeper applications with certain conditions; that is, material type A should have a minimum bending and compressive strength higher than 28 MPa and 40 MPa, respectively [29].

### 2.5. Scanning Electron Microscopy (SEM)

The morphological analysis of the fractured surface of tensile samples was conducted using SEM (Nova NanoSEM 230, FEI Co., Hillsboro, OR, USA). The fractured surface was coated with platinum using a sputter coater (EMITECH K575X, Emitech Ltd., Ashford, Kent, UK) to enhance the surface conductivity, and SEM images were taken to study the interaction between the plastic matrix and fillers.

## 3. Results and Discussion

### 3.1. Property Characterization of Raw Materials

**Figure 4:**
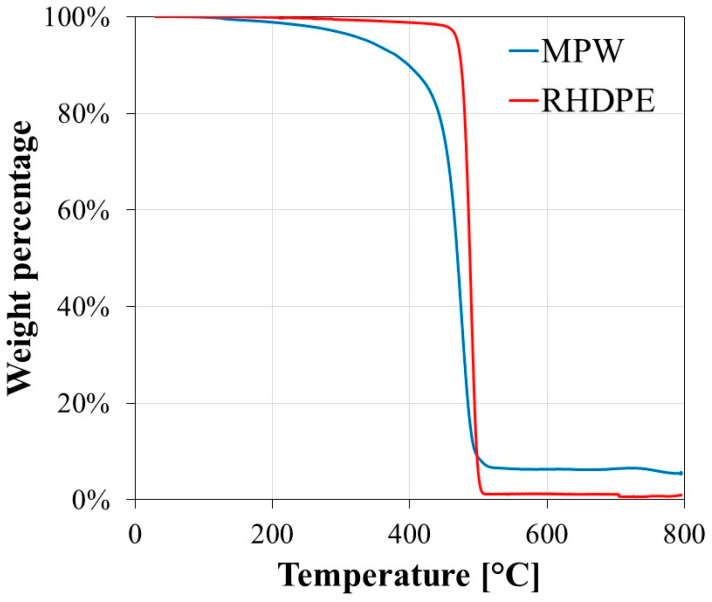
TGA results of raw plastic materials.

Figure 4 and Table 3 show the change in the weight of the specimen according to the temperature change relative to the initial mass analyzed using TGA. It can be seen that the weight changes of the plastic specimen vary depending on polymer and carbon black contents. These materials are known to be degraded in the range of 30–600 °C and 600–800 °C, respectively, and remaining residues degraded over 800 °C. The results confirm that the major components of plastic materials used in this study were polymer. Further, the TGA results indicate that MPW contains a relatively high amount of impurities.

**Figure 5:**
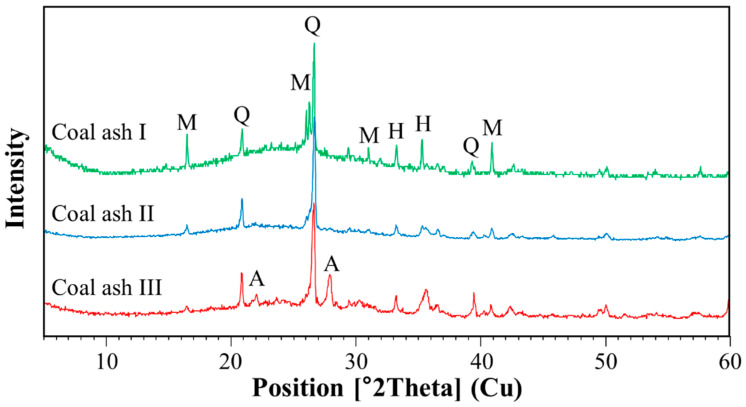
X-ray diffraction patterns for fillers: (**a**) CA I, (**b**) CA II and (**c**) CA III (M: mullite, Q: quartz, H: hematite, A: anorthite).

**Figure 6:**
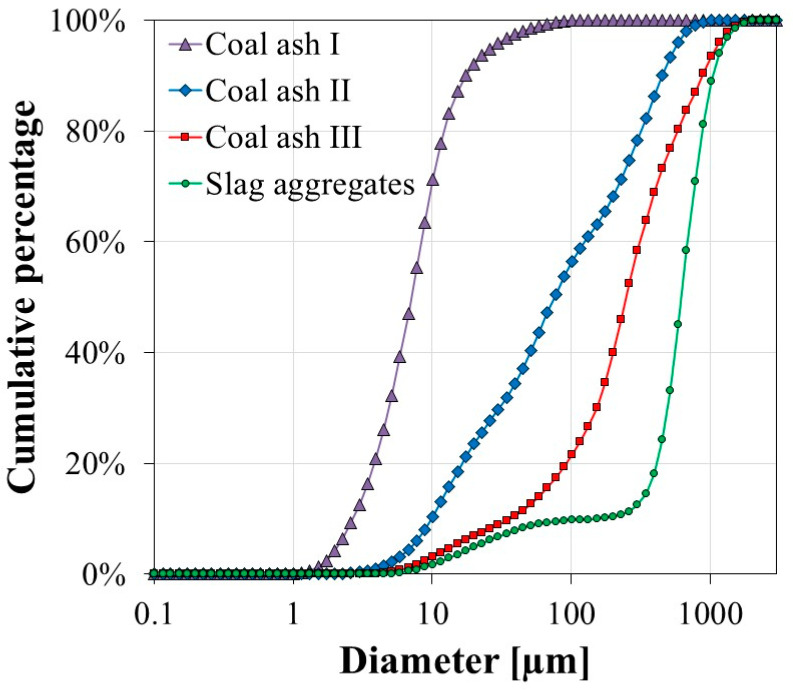
Particle size distribution of fillers (CA and slag aggregates).

The oxide compositions of plastic materials, antistatic agent and fillers determined by XRF are tabulated in Table 4 and Table 5. CAs were found to contain about 50% of SiO_2_, whereas slag aggregates contained higher amounts of CaO and Fe_2_O_3_. As shown in Figure 5, XRD analysis of CA identified an amorphous phase in addition to the presence of crystalline phases, such as quartz (Q), mullite (M), hematite (H) and anorthite (A, only for CA III). Suthar and Aggarwal [30] also observed the crystalline phases of CA. Figure 6 shows the particle size distribution of fillers. As indicated from the XRF analysis and particle size information, the oxide content in CA particles does not depend on particle size.

The physical properties of polymers are closely related to the length of the polymer chain or, equivalently, the molecular weight [31]. The molecular weight distributions of plastic materials were analyzed by GPC in terms of the number average molecular weight (M_n_), weight-average molecular weight (M_w_) and PDI (M_w_/M_n_), and the results are tabulated in Table 6. The M_n_ and M_w_ of the MPW were lower than those of the RHDPE by 27% and 19%, respectively, while the PDI were similar. The M_w_ and PDI are influential parameters for flow properties [32], and the results imply that both plastic materials may have good flowabilities.

### 3.2. Mechanical Properties

**Figure 7:**
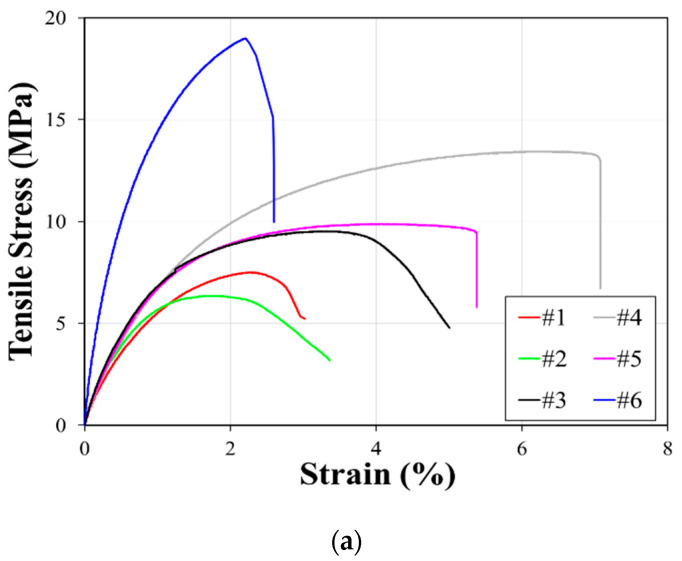

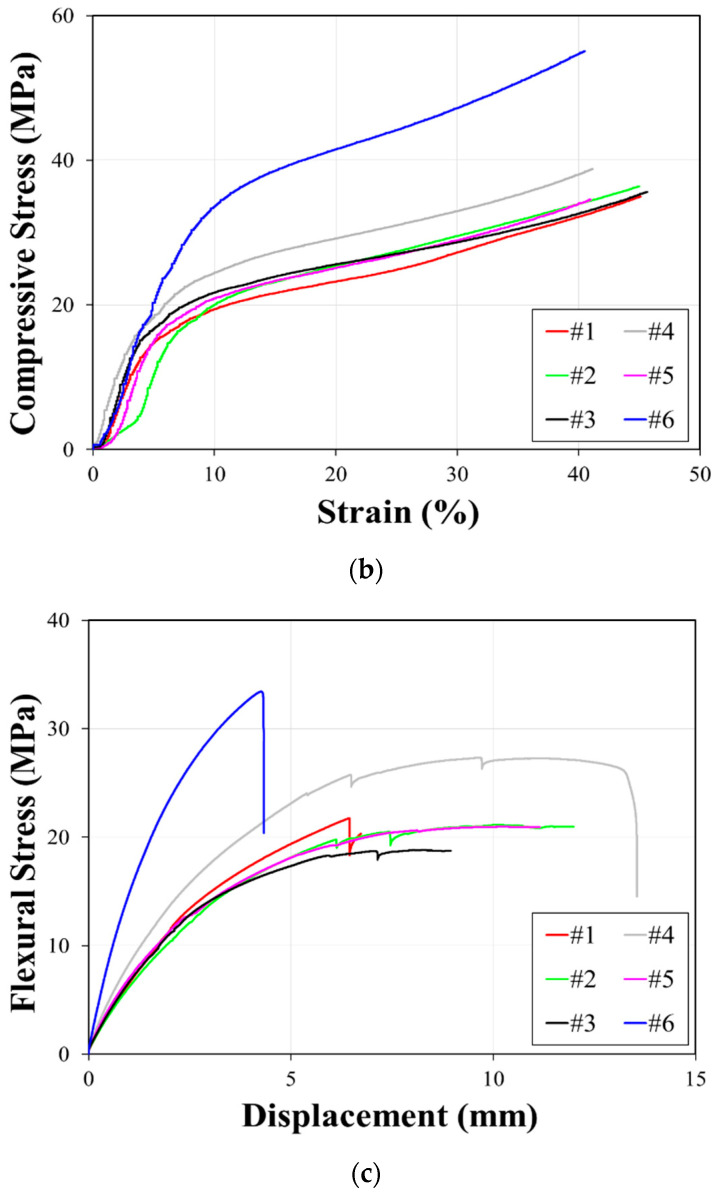
Mechanical responses of particulate composites: (**a**) tensile stress–strain relationships, (**b**) compressive stress–strain relationships and (**c**) flexural stress–displacement relationships.

**Figure 8:**
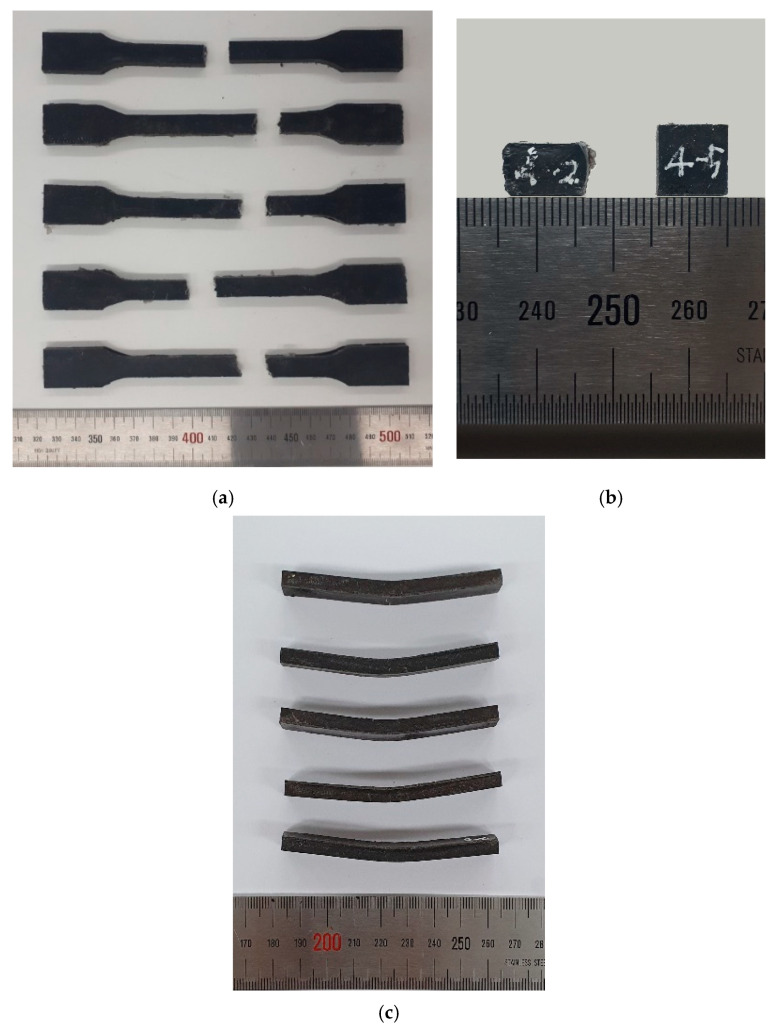
Examples of tested specimens: (**a**) tensile test, (**b**) compressive test and (**c**) flexural test.

Figure 7a illustrates the representative curves of mechanical responses of the specimens under tensile loading. The tested results indicate that the tested particulate composites are all brittle under tensile loading according to the ISO classification [25]. Figure 7b depicts the representative curves of the specimens under compression and shows ductile behavior under compression. It is recommended by ISO 604:2002 [27] and Bierögel and Grellmann [33] that compressive stress at certain strain can be used for the representative material property under compression for ductile plastics for cases in which the materials do not break or exhibit a yield point. In this study, the compressive stress at 30% strain was used as a fair comparison for the material property under compression. Figure 7c shows the representative curves of the relationship between flexural stress and displacement. Table 7 and Table 8 provide numerical summaries of the mechanical test results of the tested composites under three different loading cases. Figure 8 shows examples of tested specimens after the mechanical tests.

The particulate composites reinforced with CA (series two to four) showed mostly better mechanical performances compared with the composites reinforced with CCF (series one) or slag aggregates (series five). In addition, the composites with smaller particle-sized CA showed enhanced mechanical properties. Specifically, although the series two and three showed comparable material responses under compressive and flexural loading, the series with the smaller particle size (series three) gave higher tensile responses compared with the composites reinforced with the larger particle size (series two). Furthermore, the composite reinforced with the smallest particles (series four) showed the best mechanical responses among the tested composites in this study with five types of fillers. This result would be mostly attributed to the better interfacial adhesion between filler and matrix, and thus an enhanced load transfer. It can therefore be concluded that the mechanical properties of particulate plastic-based composites depend significantly on the particle size of the inorganic fillers, considering the facts that the chemical compositions of three types of CA are similar, and the particle sizes of CA are smaller than that of the slag aggregate. The experimental findings comply with the literature that the mechanical properties improve as the particle size of fillers decreases in inorganic particle-reinforced polymeric composites [11,34]. Parvaiz et al. [35] also concluded that the smaller particle-sized fillers substantially increased the specific area of the filler, which could effectively improve the load transfer between polymer matrix and reinforcing fillers.

The effects of filler content on the mechanical performance were evaluated using the smallest CA filler by increasing up to 60%, and these effects can be identified by comparing the experimental results between series four and six, which indicate that as CA content increases the stiffness of the composites increases under tensile and flexural loading conditions. This finding can be explained by the rule of mixtures of composites [36], in which the stiffness of composites proportionally increases as the content of reinforcements with higher stiffness increases. It should be noted, however, that the increase in reinforcement content resulted in the increase in brittleness of the composites. In addition, the tensile elastic moduli evaluated in this research are similar to the ranges found in comparative studies in the literature, such as fly ash particle-reinforced virgin HDPE matrix composites [37] and fly ash particle-reinforced RHDPE matrix composites [22].

Based on the series of mechanical tests performed, it was identified that the recycled plastic-based composite reinforced with 60% CA filler (series 6) met the minimum requirements in terms of flexural and compressive strength for railway plastic sleepers suggested by the international standard [29]. All the tested specimens showed greater strength than the ISO requirements. Further, this research confirms the possibility that industrial by-products and municipal waste can be effectively reused as primary materials for railway plastic sleepers.

### 3.3. Microstructure Characterizations

**Figure 9:**
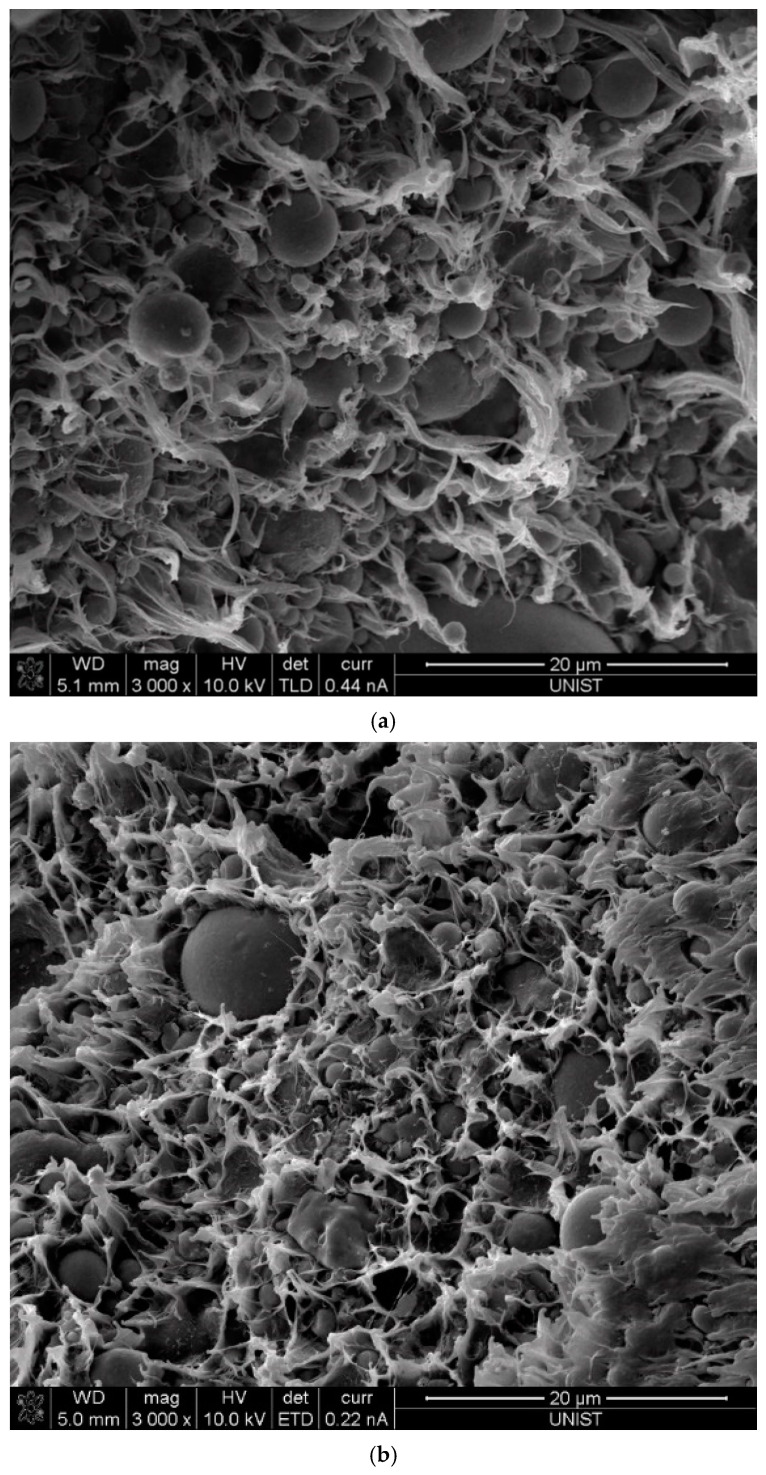
SEM images of the fractured surface (**a**) #1 and (**b**) #6.

SEM analysis was carried out to examine the morphology of particulate composites with various types of fillers. Figure 9 shows the representative SEM images of tensile fractured surfaces of the tested particulate composites, which clearly illustrate the considerable ductile fibrillary deformation of the plastic matrix. In addition, the fillers are smoothly encapsulated in the plastic matrix. It was also observed that no agglomerated fillers were identified in the SEM images even with a content of 60% CA. It can be concluded that the enhanced mechanical properties of the tested particulate composites are also attributed by the well distributed fillers and the smooth encapsulation of the fillers in the matrix.

## 4. Conclusions

This experimental study investigated the mechanical properties of inorganic particle-reinforced recycled plastic-based composites for railway sleepers, compared with the minimum requirements suggested by the international standard. Two types of recycled plastic materials and five types of fillers were used in the composites as matrices and reinforcements, respectively. The composites were prepared by a twin-screw extruder and mechanically evaluated under compressive, tensile and flexural loading conditions. The key observations and findings of this research can be summarized as follows:

1. The TGA results confirm that the major components of the recycled plastic materials used in this study are polymer, and MPW contains a relatively high amount of impurities compared with RHDPE.
2. The series of experimental results highlight that the mechanical properties of particulate plastic-based composites depend considerably on the particle size of the inorganic fillers. The composite reinforced with the smallest particles (CA I) showed the best mechanical responses in this study because of an increase in the specific area of the filler, which could effectively improve the load transfer between a polymer matrix and reinforcing fillers.
3. The SEM analysis demonstrates that the enhanced mechanical properties of the tested particulate composites are evidenced by the well distributed fillers and the smooth encapsulation of the fillers in the matrix.
4. It is identified from the series of mechanical tests that the recycle plastic-based composite reinforced with 60% of CA filler meets the minimum requirements suggested by the international standard. These research findings highlight that industrial by-products and municipal waste can be effectively reused in large quantities as primary materials for railway plastic sleepers.

It is hoped that the experimental findings in this research will provide basic information for developing sustainable railway plastic sleepers. Additional research is needed to characterize the material behavior of the developed composites at a structural level under railway loading conditions.

## Figures and Tables

**Table 1:** Density of raw materials (unit: g/cm^3^).

MPW	RHDPE	CA I	CA II	CA III	CCF	Antistatic Agent	Slag Aggregates
1.42	1.08	3.41	2.40	2.48	2.49	1.23	3.41

**Table 2:** Mix proportions (%).

Series	MPW	RHDPE	CCF	CA I	CA II	CA III	Slag Aggregates	Antistatic Agent	Other Additives
#1	30	40	30						
#2	30	40				30			
#3	30	40			30				
#4	30	40		30					
#5	30	40					30		
#6	12	20		60				4	4

**Table 3:** TGA analysis of raw plastic materials.

Materials	MPW	RHDPE
Polymer (30–600 °C)	93.7%	98.8%
Carbon black (600–800 °C)	1.0%	0.2%
Ash (Residue at 800 °C)	5.3%	1.0%

**Table 4:** XRF results of raw plastic materials and antistatic agent.

	CaO	SO_3_	Fe_2_O_3_	TiO_2_	Cl	Br	ZnO	PbO	CuO
MPW	17.0%	0%	16.4%	50.2%	13.4%	0%	1.0%	0%	0.5%
RHDPE	26.1%	3.1%	6.0%	45.4%	0%	10.7%	4.7%	2.4%	1.6%
Antistatic agent	25.1%	67.7%	7.2%	-	-	-	-	-	-

**Table 5:** XRF results of fillers.

	SiO_2_	Al_2_O_3_	Fe_2_O_3_	CaO	MgO	K_2_O	TiO_2_	Na_2_O
CA I	49.3%	26.8%	6.36%	6.44%	2.37%	1.43%	1.58%	2.12%
CA II	53.7%	21.4%	12.3%	4.91%	2.37%	1.73%	1.22%	1.32%
CA III	49.3%	19.4%	15.9%	8.43%	1.85%	1.49%	1.33%	0.77%
Slag aggregates	14.4%	2.02%	26.0%	44.3%	6.76%	0.13%	0.54%	0.07%

**Table 6:** Molecular weight of raw plastic materials obtained from gel permeation chromatography (GPC) analysis.

	M_n_	M_w_	PDI (M_w_/M_n_)
MPW	14,960	301,070	20.1
RHDPE	20,573	373,710	18.2

**Table 7:** Summary of tensile properties of the tested composites.

Series	Tensile Stress at Max. Load (MPa)	Strain at Break (%)	Modulus (MPa)
#1	6.8 ± 3.1	2.7 ± 1.4	755 ± 151
#2	6.9 ± 2.1	3.2 ± 2.4	994 ± 239
#3	9.0 ± 1.3	4.1 ± 2.1	955 ± 100
#4	13.1 ± 0.4	7.1 ± 3.5	974 ± 113
#5	9.6 ± 0.7	5.5 ± 0.7	1049 ± 131
#6	19.0 ± 0.7	2.5 ± 0.4	2281 ± 105

**Table 8:** Summary of the mechanical test results of the tested composites under compressive and flexural loading.

Series	Compressive Stress at 30% strain (MPa)	Flexural Strength (MPa)
#1	27.1 ± 1.6	19.9 ± 2.0
#2	29.0 ± 1.6	18.8 ± 1.8
#3	28.1 ± 2.0	19.0 ± 1.3
#4	32.4 ± 5.1	26.7 ± 2.5
#5	29.0 ± 2.3	19.6 ± 3.0
#6	47.1 ± 1.0	33.0 ± 0.6
